# Information and counselling on post-mortem reproduction: a Delphi study with ART specialists

**DOI:** 10.1007/s10815-026-03880-8

**Published:** 2026-04-23

**Authors:** Maria Rodrigues, Margarida Silvestre, Mariana V. Martins

**Affiliations:** 1 1Faculty of Psychology and Education Sciences, University of Porto, Porto, Portugal; 2 Faculty of Medicine, University of Coimbra, Coimbra, Portugal; 3 Medical Sciences Department, University of Aveiro, Aveiro, Portugal; 4 Centre of Psychology at University of Porto, Porto, Portugal

**Keywords:** Post-mortem reproduction, ART, IUI, Delphi study, Psychosocial counselling

## Abstract

**Purpose:**

To explore what issues should be addressed in terms of information, psychosocial counselling, and future guidelines for individuals considering post-mortem ART/IUI.

**Methods:**

A three-round Delphi study was conducted (December 2024 to August 2025) among international ART specialists (embryologists, physicians, nurses, and clinical psychologists). The study was disseminated via the mailing lists of the European Society of Human Reproduction and Embryology (ESHRE) and the Portuguese Society of Reproductive Medicine (SPMR), with the participation of 39 members. In round one, an open-ended question elicited expert perspectives on key issues in information provision, psychosocial counselling, and future guidance for post-mortem ART/IUI. Responses were subjected to qualitative thematic analysis. In round two, participants rated their agreement with each item using a five-point Likert scale. Consensus was reached if ≥ 70% of participants agreed/disagreed with a statement. In round three, prioritisation of items was performed.

**Results:**

The most important points identified were the need to safeguard legal and procedural aspects, the need to ensure prior written consent from the deceased partner, and the need to integrate psychological counselling throughout the post-mortem ART/IUI process. Areas of divergence were also identified, in relation to bereavement periods, family involvement, and long-term psychosocial support. Emerging themes underrepresented in the literature were also identified, including the psychological impact on healthcare professionals and broader social and cultural considerations.

**Conclusions:**

These findings underscore the need for multidisciplinary, consensus-informed guidance to support clinical practice in post-mortem ART/IUI. In the same way, this study highlights critical gaps in legal clarity, psychological support, and family-centred and child-centred considerations.

## Introduction

What ethical, clinical, and legal challenges arise when a reproductive decision is made after the death of one member of a couple? Although the birth of children after the death of a parent is historically documented, particularly in post-war contexts where children were born following paternal death during conflict (e.g. [1]), post-mortem ART/IUI (Assisted Reproductive Technology**/**Intrauterine Insemination) differs from these situations by constituting an active decision to continue a parental project previously established by the couple [2]. This practice represents an emerging and complex area of Medically Assisted Reproduction (MAR), confronting healthcare professionals, families, and particularly the surviving partner with deeply complex ethical, emotional, and clinical dilemmas [3]. In the absence of robust empirical evidence and standardised clinical pathways, professional judgement and expert consensus play a particularly central role in informing decision-making in this field.

The motivations associated with post-mortem ART/IUI are diverse and include the continuation of a parental project, the desire to maintain a symbolic, physical, and psychological connection with the deceased partner, and difficulties in processing grief [4]. These motivations raise important ethical and psychological questions, particularly regarding respect for the autonomy of the deceased, the welfare of the future child, and potential family conflicts [2].

Advances in gamete and embryo cryopreservation have enabled the clinical feasibility of post-mortem ART/IUI, which currently encompasses three main modalities: (1) use of genetic material cryopreserved prior to death (e.g. sperm, oocytes, or embryos), (2) post-mortem gamete extraction, and (3) transfer of embryos cryopreserved prior to death [5]. International regulation remains highly heterogeneous, with the practice authorised in some countries—generally conditional upon explicit written consent—while prohibited in others [6]. Key legal milestones emerged in the 1980 s and 1990 s, notably the Parpalaix case (France, 1984) and the Blood case (United Kingdom, 1997), which brought judicial attention to issues of consent, reproductive autonomy, and post-mortem use of gametes [7]. However, these cases did not result in harmonised regulation, partly due to the absence of empirical data on the psychosocial development of children conceived through post-mortem ART/IUI and to differing cultural, ethical, and legal interpretations of the parental project [3].

This regulatory diversity highlights the limited capacity of legislation alone to address the clinical and psychosocial dimensions of post-mortem ART/IUI. Decision-making frequently extends beyond legal eligibility and requires integration of ethical reflection and psychosocial assessment. Reflecting this complexity, the ESHRE Task Force on Ethics and Law [3] recommended that post-mortem techniques should be permitted only with the explicit consent of the deceased and following a minimum one-year waiting period.

Existing literature has primarily focused on core ethical principles related to post-mortem ART/IUI, including respect for the autonomy of the deceased, concern for the welfare of the future child, and recognition of psychological vulnerability during bereavement [4]. Nevertheless, substantial gaps persist. These include the absence of homogeneous legal and clinical guidelines and a lack of consensus regarding the mandatory role, timing, and scope of psychological counselling in post-mortem ART/IUI decision-making.

Healthcare professionals therefore occupy a pivotal position in this process. Empirical studies reveal considerable variation in professional attitudes toward post-mortem ART/IUI, while consistently emphasising the importance of informed consent, psychological counselling, and careful risk assessment [2, 8, 9]. These variations reflect the influence of tacit professional knowledge and divergent normative frameworks that are rarely formalised within existing guidance yet strongly shape clinical practice. At the same time, uncertainty remains regarding how information provision and psychosocial support should be structured and delivered across the different stages of decision-making. Counselling has been described as potentially encompassing pre-mortem psychological support, post-mortem counselling related to gamete retrieval, assessment of emotional readiness, and exploration of ethical and psychological dilemmas inherent to post-mortem ART/IUI [4], but clear guidance on its implementation remains limited.

The present study seeks to contribute to this body of knowledge by systematically capturing and synthesising the perspectives of an international panel of experts in assisted reproductive technology (ART). Using a structured expert-consensus approach, the study aims to identify areas of consensus, divergence, and priority in relation to post-mortem ART/IUI, particularly in domains where empirical evidence remains scarce and clinical practice is heterogeneous. This contribution is especially timely given recent legislative developments in several countries [10], the increasing number of requests for post-mortem procedures [11], and the continued absence of clear and harmonised clinical guidelines. By offering a cross-national perspective, this study seeks to inform future guideline development, policy deliberations, and the construction of a more transparent, ethically grounded, and psychosocially informed framework for decision-making in post-mortem ART/IUI.

## Method

### Design

Given the ethical, legal, and psychosocial complexity of post-mortem ART/IUI, and the absence of harmonised clinical guidelines, a Delphi approach was considered particularly appropriate to capture and synthesise multidisciplinary expert judgement in this field. A Delphi study is a structured group communication process designed to systematically elicit, refine, and prioritise expert opinion on a specific issue, particularly in contexts characterised by complexity, ethical sensitivity, and limited empirical evidence [12]. The Delphi approach is particularly suited to contexts in which empirical evidence is limited, as it supports the systematic organisation and prioritisation of expert knowledge across successive rounds [13].

Three rounds were planned, with three iterations usually being sufficient to provide readily available information and reach a consensus in most cases [12]. In the first round, an open-ended questionnaire was distributed to gather expert perspectives on key issues related to information provision and psychosocial counselling in decision-making regarding post-mortem ART/IUI. Given the scarcity of literature in this field, an exploratory open format was selected as the most appropriate starting point. At the beginning of Round I, participants completed a brief demographic questionnaire covering professional background and nationality. They were then invited to answer a single open-ended question: “Please list the 10 main issues that should be addressed in terms of information, psychosocial counselling, and future guidelines for individuals considering post-mortem ART/IUI.” Responses were analysed using inductive thematic analysis following Braun and Clarke’s protocol [14]. Statements were coded and grouped into latent themes based on shared underlying meanings, resulting in a consolidated list of distinct statements to be evaluated in subsequent rounds. In Round II, participants received a questionnaire comprising the consolidated list of statements generated in Round I. They were asked to rate their level of agreement with each item using a five-point Likert scale ranging from “strongly disagree” to “strongly agree”. Quantitative analysis included the calculation of medians and percentage agreement to assess whether items met the predefined threshold for agreement.

Round III included only the items that achieved agreement (> 70%) in Round II. Participants were asked to select the statements they considered most relevant and to rank these according to importance. Quantitative data analysis methods were applied to analyse responses collected in Rounds II and III. An inverted scoring system was applied, allowing for the calculation of each item’s mean ranking position and the frequency with which it appeared among the highest-priority selections. In the inverted scoring system, lower mean ranking scores indicate higher priority, as items ranked closer to first position receive lower numerical values. This process enabled the identification of statements reflecting the strongest expert consensus regarding priorities for psychosocial counselling and guideline development.

### Consensus

**Fig. 1:**
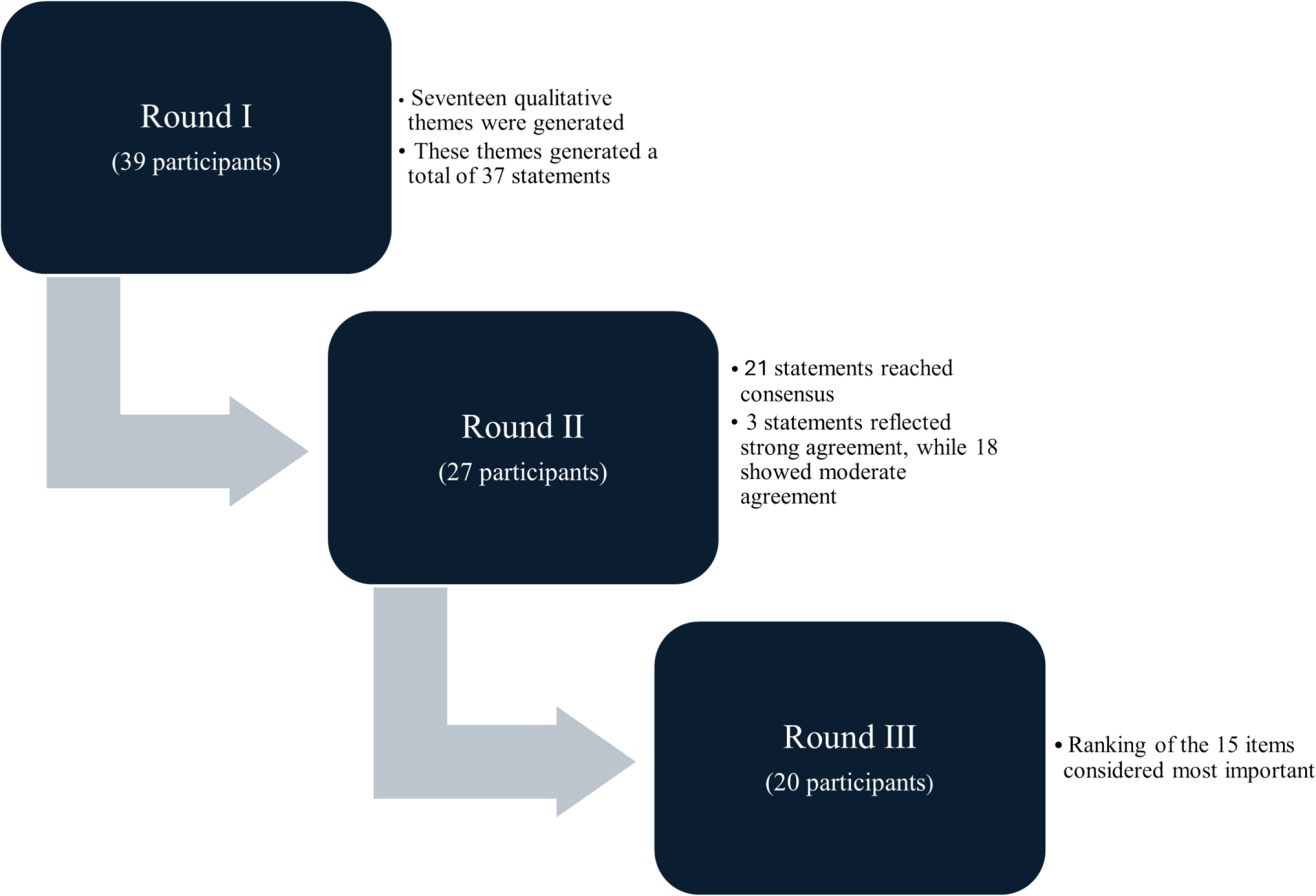
The Delphi process

The definition and criteria for consensus and agreement were established a priori. Consensus was understood as collective agreement [13]. Agreement, in turn, was defined quantitatively, requiring 70% of the panel to rate each statement as “agree” or “strongly agree” on a five-point Likert scale, in line with methodological evidence from Delphi studies in the health sciences, where consensus is most frequently defined using percentage agreement with cut-offs typically ranging between 70 and 80%, and values below 70% are considered uncommon [15]. Consensus and agreement were not considered sufficient criteria for terminating the Delphi process, as areas of divergence were also regarded as informative for understanding the complexity of the topic and identifying unresolved or contested domains within post-mortem ART/IUI decision-making. The consensus process is depicted in Fig. 1.

### Procedure

This study was approved by the Ethics Committee of the Faculty of Psychology and Education Sciences of the University of Porto (Ref. 2024-10-05). Participants received written information about the study and could contact the principal investigator if they had any questions. Consent was obtained before completion of the first-round questionnaire. All questionnaires were distributed via email and collected and stored using Qualtrics software. The questionnaires were anonymous and designed to ensure confidentiality and to protect participant identity throughout all stages of data processing.

### Participants

Eligibility criteria were defined prior to participant recruitment and focused on identifying professionals with recognised expertise in ART. Experts were eligible if they were one of the following professionals with experience in ART: embryologist, medical specialist, psychologist or other professional with recognised training in counselling (e.g. social workers), or nurse. A deliberate attempt was made to select an international expert panel to capture a wide range of perspectives resulting from different legal frameworks, cultural contexts, and clinical practices. In recognition of the absence of harmonised international regulation, the assembly of an international expert panel was deemed essential to strengthen the robustness and relevance of the consensus process.

International collaboration was sought from several professional organisations: the American Society for Reproductive Medicine (ASMR), the Brazilian Association of Assisted Reproduction (SBRA), the Brazilian Society of Human Reproduction (SBRH), the British Fertility Society (BFS), the Fertility Society of Australia and New Zealand (FSANZ), the European Society for Human Reproduction and Embryology (ESHRE), and the Portuguese Society for Reproductive Medicine (SPMR). Only ESHRE and SPMR agreed to circulate the email invitation. Questionnaires were provided in Portuguese for SPMR members (*n* = 232) and in English for ESHRE members (*n* = 9445) to ensure full accessibility. In each round of the Delphi method, participants were contacted by email on three occasions, including two reminder messages, to maximise response rates. The first round took place in January, the second in May, and the third in July 2025. This staggered data collection allowed sufficient time to analyse and refine items between rounds, in accordance with recommended Delphi procedures.

## Results

### Round I

In Round I, the questionnaire was opened 168 times, of which 129 entries contained no response to the central question. Thirty-nine (*n* = 39) responses were therefore considered complete. The expert panel consisted of 18 embryologists (46.2%), 16 medical specialists (41%), 3 psychologists (7.7%), and 2 nurses (5.1%). Of the 39 experts, 13 (33.3%) were Portuguese. Three (11.5%) were Indian, 2 (7.7%) were Greek, 2 (7.7%) English, 2 (7.7%) Dutch, 2 (7.7%) American, and single representatives (3.8%) from Romania, Turkey, Switzerland, France, Montenegro, Kazakhstan, Italy, Norway, Belgium, Israel, Germany, Spain, and Barbados.

**Fig. 2:**
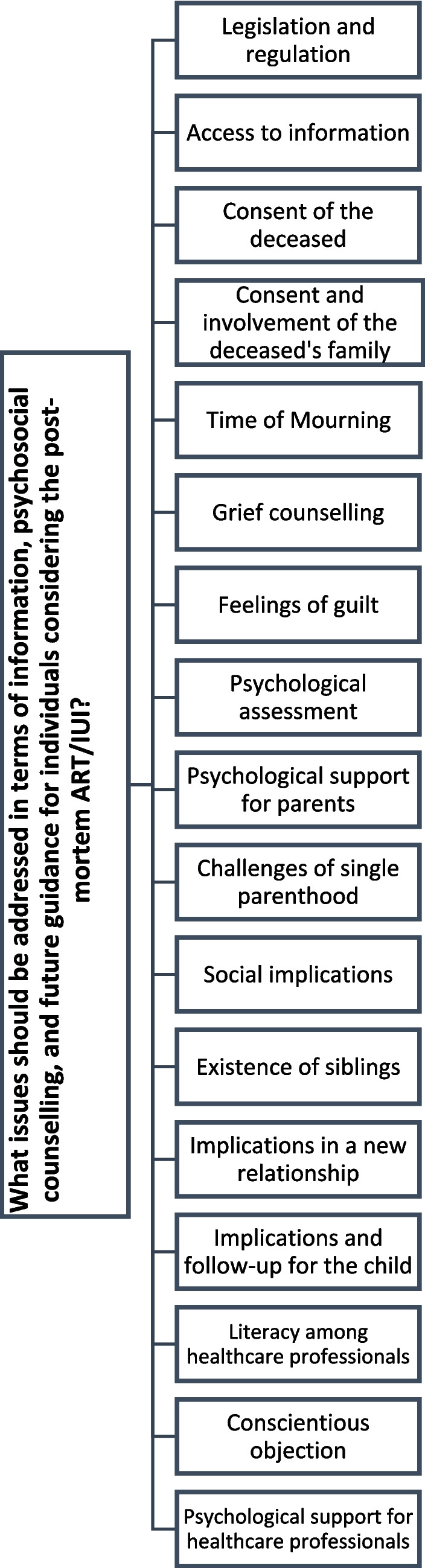
Thematic domains identified in Round I regarding information provision, psychosocial counselling, and future guidance in post-mortem ART/IUI

All responses from Round I were coded using thematic analysis, with individual codes grouped into broader themes. Seventeen qualitative themes relevant to information provision and psychological counselling in post-mortem ART/IUI were generated. These themes encompassed: legislation and regulatory requirements; necessary information regarding the request for post-mortem ART/IUI, including technical clarification; written consent from the deceased authorising post-mortem use of genetic material; the role of the deceased’s family in authorising use of the material and potential involvement in the child’s life; period of bereavement prior to initiating the procedure; provision of psychological support and counselling during the mourning process; feelings of guilt arising during decision-making; the need for psychological assessment before insemination; psychological support for the couple (before one member is deceased); challenges inherent to single parenthood; social implications associated with post-mortem ART/IUI; impact on pre-existing siblings; implications for future romantic relationships; psychological implications for children conceived through post-mortem ART/IUI across development; professional training and literacy on post-mortem ART/IUI; right to conscientious objection; and the provision of psychological support to health professionals. These themes generated a total of 37 statements, which were subsequently presented to experts in Round II. Figure 2 presents a summary of the identified themes.

### Round II

The Round II questionnaire was completed by 27 experts, corresponding to a response rate of 69.2%. The professional composition and geographical distribution of Round II participants were comparable to Round I, although slightly fewer psychologists and non-European participants were represented. In Round II, all 37 statements were presented for evaluation of agreement. Table 1 summarises levels of agreement, medians, and interquartile ranges for each statement, as well as those meeting the predefined consensus threshold (> 70%). Of these, three statements reflected strong agreement (> 90%), while 18 showed moderate agreement (> 70% and < 90%). In contrast, 14 statements did not achieve agreement (< 70%), 9 of which showed weak agreement (> 50% and < 70%). The items with the highest level of agreement (95.8%) were: “Psychological counselling can help to clarify whether the decision to undergo the procedure is being made in a conscious and informed manner”, “The child’s inheritance and succession rights must be legally guaranteed” and “The lack of adequate training may compromise the quality of care provided to people requesting the procedure”. The item with the lowest level of agreement was “An assessment of the family support and socioeconomic conditions of the beneficiary partner should be carried out”. This item obtained the lowest level of agreement (33.3%), indicating substantial divergence in expert opinion.

**Table 1:** Levels of agreement among ART specialists regarding information provision, psychosocial counselling, and guidance in post-mortem ART/IUI (Round II)

	M (SD)	Median	Inter-quartile range		Agreement %
Topic 1: Legislation and regulation					
Guidelines should be developed for healthcare professionals in assisted reproductive technology (ART) to support decision-making and advice in cases of post-mortem insemination	4.63 (0.93)	5.0		0	88.9%
Topic 2: Access to Information					
Information about the possibility of performing post-mortem insemination should be widely disseminated so that couples can consider it before a potential loss	3.92 (1.32)	4.5		2.0	69.2%
Healthcare institutions should provide clear guidance on the steps required to request post-mortem insemination	4.42 (1.03)	5.0		1.0	80.8%
In response to any questions raised by the patient(s), healthcare professionals should provide detailed information about the procedures and success rates of post-mortem insemination	4.46 (0.91)	5.0		1.0	92.3%
Topic 3: Consent of the deceased					
It is essential that there be written informed consent from the deceased partner attesting to their wish for the beneficiary to use their genetic material post-mortem	4.46 (1.1)	5.0		0.8	88.5%
Topic 4: Consent and Involvement of the Deceased's Family					
The decision to perform post-mortem insemination should not be solely the beneficiary's – other direct relatives of the deceased should be involved in the process	2.84 (1.41)	3.0		2.0	48.0%
The immediate family members of the deceased partner should have the right to be involved in the life and development of the child	3.80 (1.12)	4.0		2.0	64.0%
Topic 5: Time for Mourning					
There must be a minimum mourning period before allowing a request for post-mortem insemination	4.00 (1.38)	5.0		2.0	72.0%
There must be at least one year of bereavement before the beneficiary begins post-mortem insemination treatments	3.64 (1.41)	4.0		2.0	60.0%
Topic 6: Grief Counselling					
The psychological support provided to the beneficiary should include the provision of socio-emotional tools to manage the emotional distress associated with the loss of her partner	4.60 (0.71)	5.0		1.0	88.0%
The assisted reproductive technology (ART) centre must ensure that the recipient makes an informed decision in a bereaved situation, including clarifying that post-mortem insemination will not replace the deceased partner	4.72 (0.89)	5.0		0	92.0%
Topic 7: Feelings of Guilt					
Prior consent from the deceased may induce feelings of guilt in the beneficiary if she does not wish to undergo post-mortem insemination	3.17 (1.44)	3.5		2.0	50.0%
Regardless of whether the beneficiary ultimately decides to proceed with post-mortem insemination, psychological counselling should address any feelings of guilt that may arise	3.92 (1.14)	4.0		2.0	70..8%
Topic 8: Psychological Assessment Psychological counselling should be mandatory before post-mortem insemination is performed	4.54 (1.02)	5.0		0	87.5%
Psychological counselling can help to better clarify whether the decision to undergo the procedure is being made consciously and in an informed manner	4.88 (0.45)	5.0		0	95.8%
Topic 9: Psychological support for parents					
The partner who donates their gametes post-mortem should receive psychological counselling before giving their consent	4.13 (1.04)	4.0		1.0	83.3%
The beneficiary should receive psychological support throughout the entire process	4.44 (1.04)	5.0		1.0	88.0%
Topic 10: Challenges of single parenthood					
Post-mortem insemination can be a legitimate option for people who wish to pursue independent/solo parenting plans	3.00 (1.82)	3.0		4.0	45.8%
An assessment of the family support and socioeconomic conditions of the beneficiary partner should be carried out	2.83 (1.55)	2.5		2.5	33.3%
Topic 11: Social Implications The stigma associated with post-mortem insemination can impact the psychological well-being of the family					
	3.71 (1.27)	4.0		2.0	70.8%
The cultural and religious factors in which the future child will be integrated should be taken into account in the process	3.33 (1.58)	4.0		3.0	54.2%
Topic 12: The Existence of Siblings					
The decision to conceive a child posthumously can be influenced by the existence of other children	4.08 (0.97)	4.0		1.0	79.2%
The impact on existing siblings should be assessed before the procedure is performed	3.79 (1.29)	4.0		2.0	66.7%
Topic 13: Implications in a new relationship					
Performing post-mortem insemination can impact the recipient's future romantic relationships	3.25 (1.29)	3.0		1.3	41.7%
The existence of a new relationship should be considered when deciding whether to perform post-mortem insemination	3.79 (1.41)	4.0		2.0	70.8%
The desire to have a child with the deceased can coexist with the possibility of building a new family in the future	3.33 (1.34)	3.0		3.0	41.7%
Topic 14: Implications and psychological support for the child					
The child’s inheritance and succession rights must be legally guaranteed	4.63 (0.58)	5.0		1.0	95.8%
It would be important to elaborate on the moment of revealing the post-mortem conception to the child with the beneficiary in a consultation setting	3.96 (1.20)	4.0		2.0	70.8%
Psychological support should be provided during the development of the child conceived through post-mortem insemination	3.63 (1.21)	4.0		2.3	54.2%
Topic 15: Literacy of healthcare professionals					
Inadequate training can compromise the quality of care provided to people requesting the procedure	4.58 (0.72)	5.0		1.0	95.8%
Assisted reproductive technology (ART) professionals should receive specific training on the ethical and psychological aspects of post-mortem insemination	4.42 (0.65)	4.5		1.0	91.7%
Topic 16: Conscientious Objection					
Assisted reproductive technology (ART) professionals should have the right to refuse to perform post-mortem insemination on grounds of conscientious objection Conscientious objection should be regulated to ensure access to the procedure without compromising professional ethics	3.75 (1.54) 4.25 (0.99)	4.0 4.5		2.3 1.0	70.8% 83.3%
Healthcare institutions may establish their own guidelines for performing post-mortem insemination	2.92 (1.56)	3.0		3.0	41.7%
Topic 17: Psychological support for healthcare professionals					
Psychological support should be made available to professionals working in this field	4.00 (1.14)	4.0		2.0	66.7%

### Round III

The Round III questionnaire was completed by 20 experts, corresponding to a 51.3% response rate. The composition of Round III participants remained broadly similar, although further reduction in sample size may have influenced representativeness. Experts were first asked to select the 15 most important statements among the 21 that had reached consensus in Round II. Table 2 presents the frequency and ranking of the prioritised statements. The statement “Guidelines should be developed for healthcare professionals in ART to support decision-making and counselling in cases of post-mortem ART/IUI” was selected as important by all participants. In the ranking of priorities, this statement also achieved the highest popularity score and the highest mean ranking, indicating strong consensus regarding its centrality for clinical practice.

**Table 2:** Prioritisation of consensus statements by ART specialists regarding key elements of post-mortem ART/IUI practice (Round III)

	Frequency (%)	Sum	Mean
Guidelines should be developed for healthcare professionals in assisted reproductive technology (ART) to support decision-making and advice in cases of post-mortem insemination	20 (100%)	251	13.21
It is essential that there be written informed consent from the deceased partner attesting to their wish for the beneficiary to use their genetic material post-mortem	18 (90%)	213	12.53
Healthcare institutions should provide clear guidance on the steps required to request post-mortem insemination	16 (80%)	181	12.07
There must be a minimum bereavement period before allowing a request for post-mortem insemination	16 (80%)	161	10.73
Assisted reproductive technology (ART) professionals should receive specific training on the ethical and psychological aspects of post-mortem insemination	16 (80%)	74	4.63
In response to any questions raised by the patient(s), healthcare professionals should provide detailed information about the procedures and success rates of post-mortem insemination	15 (75%)	162	10.8
Psychological support for the beneficiary should include the provision of socio-emotional tools to manage the emotional distress associated with the loss of her partner	15 (75%)	127	9.07
Psychological support should be mandatory before post-mortem insemination is performed	15 (75%)	145	10.36
Conscientious objection should be regulated to ensure access to the procedure without compromising professional ethics	15 (75%)	42	2.80
The beneficiary should receive psychological support throughout the entire process	15 (75%)	114	8.14
The assisted reproductive technology (ART) centre must ensure that the recipient makes an informed decision in a bereaved situation, including clarifying that post-mortem insemination will not replace the deceased partner	14 (70%)	118	9.07
The partner who donates their gametes post-mortem should receive psychological counselling before giving their consent	14 (70%)	113	8.69
The child's inheritance and succession rights must be legally guaranteed	14 (70%)	82	6.31
The lack of adequate training can compromise the quality of care provided to people requesting the procedure	14 (70%)	67	4.79
The existence of a new relationship should be considered when deciding whether to perform post-mortem insemination	13 (65%)	63	5.25
It would be important to plan the moment of revealing the post-mortem conception to the child with the beneficiary in a consultation setting	13 (65%)	48	4.0
Assisted reproductive technology (ART) professionals should have the right to refuse to perform post-mortem insemination on grounds of conscientious objection	13 (65%)	70	5.39
Psychological counselling can help to better clarify whether the decision to undergo the procedure is being made consciously and in an informed manner	12 (60%)	94	8.55
The stigma associated with post-mortem insemination can impact the psychological well-being of the family	12 (60%)	48	4.36
The decision to conceive a child posthumously can be influenced by the existence of other children	12 (60%)	54	4.91
Regardless of whether the beneficiary ultimately decides to undergo post-mortem insemination, psychological counselling should address any feelings of guilt that may arise	8 (60%)	61	7.63

## Discussion

The aim of this study was to identify and prioritise, through expert consensus, the key elements that ART specialists consider essential to address in post-mortem ART/IUI, with the broader goal of supporting the development of clinical guidelines in a context of limited empirical evidence. Across three Delphi rounds, specialists consistently emphasised legal and procedural clarity, explicit consent, and psychological counselling as foundational components of responsible post-mortem ART/IUI practice. These results contribute to the existing literature on post-mortem ART/IUI by synthesising areas of expert consensus and divergence, thereby clarifying priorities for inclusive, informed, and legally robust clinical practice in a field that remains underexplored empirically.

The qualitative analysis of the open-ended responses revealed a diversity of topics, ranging from legal and legislative issues to psychological, family, and social aspects, highlighting the multidisciplinary nature of post-mortem ART/IUI. This diversity underscores post-mortem ART/IUI as a profoundly complex reproductive decision that intersects with bereavement, autonomy, parenthood, and the child’s future identity, requiring a multidisciplinary and integrated framework of care. The breadth of themes generated in the first Delphi round underscores the importance of systematically incorporating expert perspectives when addressing post-mortem ART/IUI decision-making. Expert contributions both confirmed previously identified domains, such as conscientious objection, and broadened the scope of discussion by introducing topics that remain underrepresented in the literature, such as the need for psychological support for healthcare professionals. Although less frequently addressed in the literature, these topics raise valuable questions that broaden the understanding of post-mortem ART/IUI and highlight the psychosocial impact of this practice not only on patients and families, but also on healthcare professionals.

A central finding was the strong consensus regarding the need for written consent from the deceased partner, aligning with existing legal and ethical frameworks (e.g. [3, 16]). Written consent is viewed not only as a legal safeguard but also as a vital ethical cornerstone that legitimises the reproductive decision and mitigates potential familial or institutional conflict [2]. This emphasis suggests that, across professional backgrounds and jurisdictions, autonomy and clarity of intention are regarded as the most stabilising elements in the highly sensitive context of post-mortem ART/IUI.

Experts also affirmed the necessity of a bereavement period before initiating post-mortem ART/IUI, although no agreement emerged on its appropriate duration. This mirrors the global landscape, where legislation varies widely and often diverges from professional recommendations [6], including the recommendation of a minimum one-year mourning period proposed by the ESHRE Task Force on Ethics and Law [3]. The absence of consensus is reflected in the substantial heterogeneity of national regulations, with legal time frames ranging from very short waiting periods (e.g. one month in Estonia) to extended or undefined durations in other jurisdictions (e.g. up to 60 months in Belgium or no legally specified timeframe in countries such as Kazakhstan) [17]. Such variability suggests that temporal thresholds are frequently informed by legal, cultural, or moral considerations rather than by empirical evidence on grief trajectories. From a psychosocial perspective, a six-month period may be insufficient for emotional stabilisation following the loss of an intimate partner, particularly given the intensity of early grief reactions and the heightened vulnerability commonly observed during the first year after loss [18, 19]. At the same time, overly prolonged or undefined waiting periods may impose additional psychological burden and uncertainty, especially for women of reproductive age facing time-sensitive fertility decisions. Expert contributions both confirmed previously identified domains, such as conscientious objection, and broadened the scope of discussion by introducing topics that remain underrepresented in the literature, such as the need for psychological support for healthcare professionals.

The discrepancy in legislative harmonisation also raises concerns about reproductive tourism (e.g. [7]), whereby individuals may seek treatment in countries with fewer restrictions, thereby exacerbating inequities in access to care. In contexts where legislation is absent or ambiguous, decision-making may fall primarily under professional ethical codes and clinical judgement, raising concerns about consistency and legal certainty [20]. Experts expressed concern that when legal guidance is unclear, professionals must rely heavily on personal ethical judgment, which may contribute to variable and potentially inequitable practices. These observations strengthen the argument for consensus-informed guidelines that extend beyond fixed time frames and integrate structured psychosocial counselling with individualised assessment of emotional readiness.

Regarding information provision, experts valued transparent communication about legal and technical aspects of post-mortem ART/IUI, though some expressed reservations about broader public dissemination. This ambivalence may reflect concerns about societal stigma or child welfare, often cited as reasons for resistance [21]. The consensus on the need for professional training underscores the importance of equipping clinicians with the skills required to navigate ethically complex scenarios while maintaining the right to conscientious objection when warranted.

The right to conscientious objection and its regulation emerged as areas of expert concern, with consensus regarding its ethical legitimacy. Reproductive healthcare professionals have been confronted with these ethical tensions between personal beliefs and professional duties to provide or facilitate access to legally permitted care mostly concerning abortion [22]. However, across Europe, approaches to conscientious objection vary substantially, ranging from systems in which it is broadly protected with limited regulatory constraints (e.g. Italy and Poland) to contexts in which it is either tightly circumscribed (e.g. the UK, Norway, and Portugal) or not legally recognised within healthcare practice (e.g. Sweden and Finland) [23, 24]. This regulatory variability has direct implications for post-mortem ART/IUI practice, as weakly regulated models may compromise continuity of care, while more restrictive systems may generate ethical tension for professionals involved in complex reproductive decisions [25].

Psychological support stood out as a key element at multiple decision points. Experts emphasised that counselling should include support in decision-making in the context of grief, clarification of risks, and assessment of emotional preparedness for the social and familial implications of post-mortem ART/IUI [4]. Psychological support was also viewed as essential for helping individuals manage emotional distress and feelings of guilt associated with partner loss. This support should be continuous throughout the process and should include the partner donating the gametes. The strong consensus among specialists suggests that integrating mandatory psychological assessment and support into post-mortem ART/IUI protocols would promote safer and more ethically grounded practices. However, although the ESHRE Task Force on Ethics and Law [3] recommends that the surviving partner undergo counselling during the decision-making period, current legal frameworks often prioritise written consent and temporal restrictions, potentially limiting access to psychological support at moments of heightened emotional vulnerability.

Concerning the legal implications for the child, there was consensus among experts on the need to ensure legal protection such as inheritance and succession rights. Experts also emphasised the importance to prepare parents for future conversations with the child about the circumstances of their conception. The absence of consensus suggests uncertainty regarding the extent and timing of psychological support needs, underscoring the relevance of longitudinal research on psychosocial outcomes in post-mortem ART/IUI-conceived families [26]. Family and social dimensions were also areas of disagreement, particularly regarding the role of the deceased partner’s family and the potential impact on existing siblings—topics that are still underrepresented in the literature. However, the decision to conceive through post-mortem ART/IUI may be a risk factor for family dynamics [27]. Experts recognised that post-mortem ART/IUI may shape family dynamics in complex ways, including expectations from extended family [4]. Regarding siblings and the existence of brothers and sisters, there was consensus that this variable could influence the decision to conceive a child post-mortem, but there was no consensus on the assessment of the impact on existing siblings. There are also divergent opinions on the impact of new romantic relationships on decision-making, suggesting a need for further research into the family and social aspects of post-mortem techniques, which remain largely unexplored and lack empirical evidence to guide clinical practice. Overall, the divergence observed in family and social domains reflects the limited empirical base and highlights these areas as priorities for future research and clinical reflection.

The prioritisation exercise revealed that the legal and procedural dimension was considered a priority, suggesting that experts convey a clear concern to guarantee and ensure regulated and uniform practices. The emphasis placed on defining institutional guidelines and the existence of written consent from the deceased indicate that professionals seek to minimise legal ambiguities through uniform guidelines, reducing the risk of divergent interpretations between institutions. The psychological aspect is also prominent, highlighting the importance of structured psychological support in situations of grief and complex reproductive decisions.

The results of this study contribute to advancing knowledge about post-mortem ART/IUI by systematically capturing expert consensus and disagreement across disciplines and jurisdictions, thereby clarifying priorities for guideline development and clinical practice. The prioritisation hierarchy demonstrates the importance attributed to legal and procedural dimensions, as well as the relevance and pertinence of psychological support, outlining initial steps for defining public policies, creating future guidelines, and developing interventions that are more sensitive to the needs of the beneficiary, family, and child. In addition to their direct impact on clinical practice, these results have broad social and cultural relevance, as they raise issues such as stigma and family implications, promoting an informed debate in society. This study also stands out for the diversity of its sample, composed of specialists from various areas of expertise in the field of ART (medical specialists, embryologists, psychologists, and nurses) and from different countries and legal contexts, thereby enriching the range of perspectives. The methodology followed, based on three rigorous stages of the Delphi method, reinforces the validity of the results.

Nevertheless, there are relevant limitations to highlight. The final number of participants was affected by attrition between rounds, as the reduction in panel size potentially amplified the weight of individual opinions, thereby limiting the statistical robustness of the analysis. However, the retention rate was approximately 70% between Round I and Round II and 75% between Round II and Round III, demonstrating continuity of most participants throughout the study. Still, it was not possible to determine whether participants who completed later rounds differed systematically from those who dropped out, and therefore attrition bias cannot be excluded, particularly in the prioritisation phase. In addition, the small sample size makes it impossible to fully generalise the results, which may have contributed to the over or underestimation of consensus on some topics. Finally, the high number of initial non-responses in Round I (129 out of 168 survey openings without completion) may indicate limited engagement or response burden, raising the possibility of response bias at the recruitment stage. The uneven distribution of professional backgrounds, marked by a predominance of medical specialists and embryologists and a reduced representation of psychologists, may have shaped the prioritisation of clinical and medical considerations over psychosocial aspects, potentially affecting the relative weighting of specific themes. There was also a lack of participants from regions such as Africa, Oceania, and Latin America, which reduced global cultural and legislative diversity. Although this study aimed to include an international expert panel, recruitment relied primarily on ESHRE and SPMR mailing lists, as only these organisations agreed to disseminate the survey. This may have resulted in an overrepresentation of European perspectives and underrepresentation of other regulatory and cultural contexts, potentially limiting the generalisability of findings. As attitudes toward post-mortem ART/IUI are strongly shaped by local legal, ethical, and cultural frameworks, future studies should prioritise broader geographical inclusion to enhance cross-cultural applicability of consensus findings. Another aspect to note is that it was not possible to assess the motivations behind certain choices, such as the emergence of conscientious objection, where there were divergences but no adequate explanation of the ethical or practical principles underlying these positions. Finally, restricting Round III to items that reached the predefined agreement threshold was intended to focus the prioritisation process on areas of convergence and to reduce participant burden, a common practice in Delphi studies where questionnaires are progressively shortened across rounds to maintain engagement. However, this approach may limit the ability to assess the stability of judgments and further explore areas of disagreement, which are particularly relevant in ethically complex domains. These limitations are consistent with challenges commonly encountered in Delphi studies and should be considered when interpreting the findings.

Future research should expand the investigation to various cultural and legislative contexts to understand how local specificities influence perceptions and practices. It would also be significant to explore additional variables, such as the specialists’ personal motivations, previous experiences, or religious beliefs, which may help explain the divergences identified. Future research could thus offer a broader, more comprehensive understanding of post-mortem ART/IUI, helping to develop more complete, informed guidelines and policies tailored to the inherent ethical, psychological, and social complexities.

## Data Availability

Data is available upon reasonable request to the corresponding author.

## References

[1] Costa DL, Yetter N, DeSomer H. Intergenerational transmission of paternal trauma among US Civil War ex-POWs. Proc Natl Acad Sci. 2018;115(44):11215–20. 30322945 10.1073/pnas.1803630115 PMC6217388

[2] Quayle J. The psychologist and the posthumous assisted reproduction. Front Womens Health. 2019;4:1–6. 10.15761/FWH.1000160.

[3] ESHRE Task Force on Ethics and Law, Pennings G, de Wert G, Shenfield F, Cohen J, Devroey P et al. ESHRE Task Force on Ethics and Law 11: posthumous assisted reproduction. Hum Reprod. 2006;21:3050–3053. 10.1093/humrep/del287. 10.1093/humrep/del287 16923749

[4] Lawson AK, Zweifel JE, Klock SC. Blurring the line between life and death: a review of psychological and ethical concerns related to posthumous-assisted reproduction. Eur J Contracept Reprod Health Care. 2016;21:339–46. 10.1080/13625187.2016.1203892. 27388465 10.1080/13625187.2016.1203892

[5] Lozano JAG, Takitane J. Considerações jurídicas, éticas e médico-legais sobre a reprodução post mortem em alguns países da Ibero-América: revisão integrativa. Rev Latinoam Bioet. 2021;21:11–30. 10.18359/rlbi.4758.

[6] Jones HW, Cooke I, Kempers R, Brinsden P, Saunders D. IFFS Surveillance 2010. Fertil Steril. 2011;95:491. 10.1016/j.fertnstert.2010.08.011. 20813358 10.1016/j.fertnstert.2010.08.011

[7] Blyth E, Cameron C. The welfare of the child: an emerging issue in the regulation of assisted conception. Hum Reprod. 1998;13:2339–42. 10.1093/humrep/13.9.2339. 9806241 10.1093/humrep/13.9.2339

[8] Corrigan E, Mumford SE, Hull MG. Posthumous storage and use of sperm and embryos: survey of opinion of treatment centres. BMJ. 1996;313:24. 10.1136/bmj.313.7048.24. 8664765 10.1136/bmj.313.7048.24 PMC2351451

[9] Huang J, Li J, Xiao W, Li Z. Attitudes toward posthumous assisted reproduction in China: a multi-dimensional survey. Reprod Health. 2022;19:1–11. 10.1186/s12978-022-01423-9. 35598020 10.1186/s12978-022-01423-9 PMC9124412

[10] Turillazzi E, Morena D, Scopetti M, Fineschi V. Postmortem fertilization: New Italian government guidelines affirm the legitimacy of this procedure. Eur J Obstet Gynecol Reprod Biol X. 2024;23:100337. 10.1016/j.eurox.2024.100337. 39263392 10.1016/j.eurox.2024.100337 PMC11388668

[11] Kelsey-Sugg A, Zajac B. Posthumous sperm retrieval raises some troubling questions, but who should answer them? ABC News. 2023. https://www.abc.net.au/news/2023-08-25/posthumous-sperm-retrieval-israel-compare-to-australia/102754328.

[12] Hsu CC, Sandford B. The Delphi technique: making sense of consensus. Pract Assess Res Eval. 2007;12:10. 10.7275/pdz9-th90.

[13] Keeney S, McKenna HP, Hasson F. The Delphi technique in nursing and health research. Oxford: Wiley-Blackwell; 2011.

[14] Braun V, Clarke V. Using thematic analysis in psychology. Qual Res Psychol. 2006;3:77–101. 10.1191/1478088706qp063oa.

[15] Schifano J, Niederberger M. How Delphi studies in the health sciences find consensus: a scoping review. Syst Rev. 2025;14(1):14. 10.1186/s13643-024-02738-3. 39810238 10.1186/s13643-024-02738-3 PMC11734368

[16] Portugal. Law No. 72/2021 of 12 November 2021**.** Diário da República (Official Gazette of Portugal). Series I, No. 220. 2021:3–5. Available from: https://diariodarepublica.pt/dr/detalhe/lei/72-2021-174244807.

[17] European IVF Monitoring Consortium (EIM), for the European Society of Human Reproduction and Embryology (ESHRE); Smeenk J, Wyns C, De Geyter C, Kupka M, Bergh C, Cuevas Saiz I, De Neubourg D, Rezabek K, Tandler-Schneider A, Rugescu I, et al. ART in Europe, 2018: results generated from European registries by ESHRE. Hum Reprod. 2022;38:2321–2338.

[18] Najib A, Lorberbaum J, Kose S, Bohning D, George M. Regional brain activity in women grieving a romantic relationship breakup. Am J Psychiatry. 2004;161(12):2245–56. 10.1176/appi.ajp.161.12.2245. 15569896 10.1176/appi.ajp.161.12.2245

[19] Nielsen M, Carlsen A, Neergaard M, Bidstrup P, Guldin M. Looking beyond the mean in grief trajectories: a prospective, population-based cohort study. Social Sci Med. 2019;232:460–9. 10.1016/j.socscimed.2018.10.007. 10.1016/j.socscimed.2018.10.007 31230666

[20] Silva S, Machado H. The construction of meaning by experts and would-be parents in assisted reproductive technology. Sociol Health Illn. 2011;33:853–68. 10.1111/j.1467-9566.2010.01327.x. 21899561 10.1111/j.1467-9566.2010.01327.x

[21] Hans JD, Frey LM. American attitudes in context: posthumous use of cryopreserved gametes. J Clin Res Bioeth. 2013;S1:006. 10.4172/2155-9627.S1-006.

[22] Kuře J. Conscientious objection in health care. Ethics & Bioethics (in Central Europe). 2016;6(3–4):173–80. 10.1515/ebce-2016-0018.

[23] Zampas, C. Legal and ethical standards for protecting women’s human rights and the practice of conscientious objection in reproductive healthcare settings. Int J Gynaecol Obstet. 2013; 123. 10.1016/s0020-7292(13)60005-3. 10.1016/S0020-7292(13)60005-3 24332237

[24] Yang C. The inequity of conscientious objection: refusal of emergency contraception. Nurs Ethics. 2020;27:1408–17. 10.1177/0969733020918926. 32400261 10.1177/0969733020918926

[25] Fry-Bowers E. A matter of conscience: examining the law and policy of conscientious objection in health care. Policy Politics Nurs Pract. 2020;21:120–6. 10.1177/1527154420926156. 10.1177/1527154420926156 32443952

[26] Golombok S, Badger S. Children raised in mother-headed families from infancy: a follow-up of children of lesbian and single heterosexual mothers, at early adulthood. Hum Reprod. 2010;25(1):150–7. 10.1093/humrep/dep345. 19840989 10.1093/humrep/dep345

[27] Landau R. Planned orphanhood. Soc Sci Med. 1999;49(2):185–96. 10.1016/s0277-9536(99)00100-8. 10414828 10.1016/s0277-9536(99)00100-8

